# Supplementary material for: E-Cigarette Exposure Decreases Bone Marrow Hematopoietic Progenitor Cells
Source: Cancers (Basel). 2020 Aug 14;12(8):2292. doi: 10.3390/cancers12082292 (PMC7464997; doi:10.3390/cancers12082292)
Supplement: Supplementary file 1 [file cancers-12-02292-s001.pdf]

cancers

Supplementary Files

E-cigarette Exposure Decreases Bone Marrow Hematopoietic Progenitor Cells

Gajalakshmi Ramanathan, Brianna Craver-Hoover, Rebecca J. Arechavala, David A. Herman, Jane H. Chen, Hew Yeng Lai, Samantha R. Renusch, Michael T. Kleinman and Angela G. Fleischman

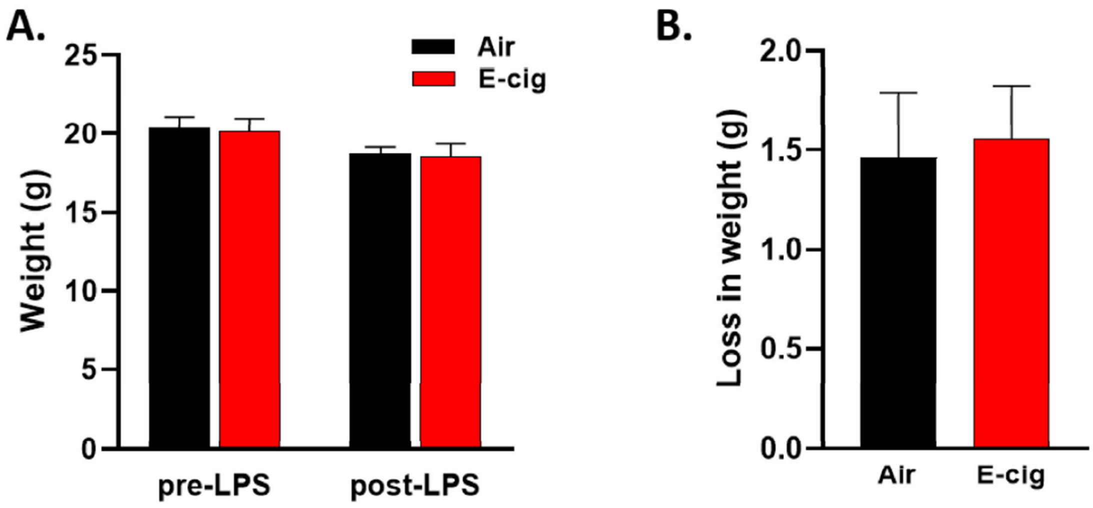

Figure S1. Systemic LPS leads to loss in body weight. (A) Weight of air and E-cig exposed before and16 hours after LPS injection. (B) Change in body after in air and E-cig exposure groups 16 hours after LPS treatment. Data are shown as mean±SEM. n=6-8 mice per group.

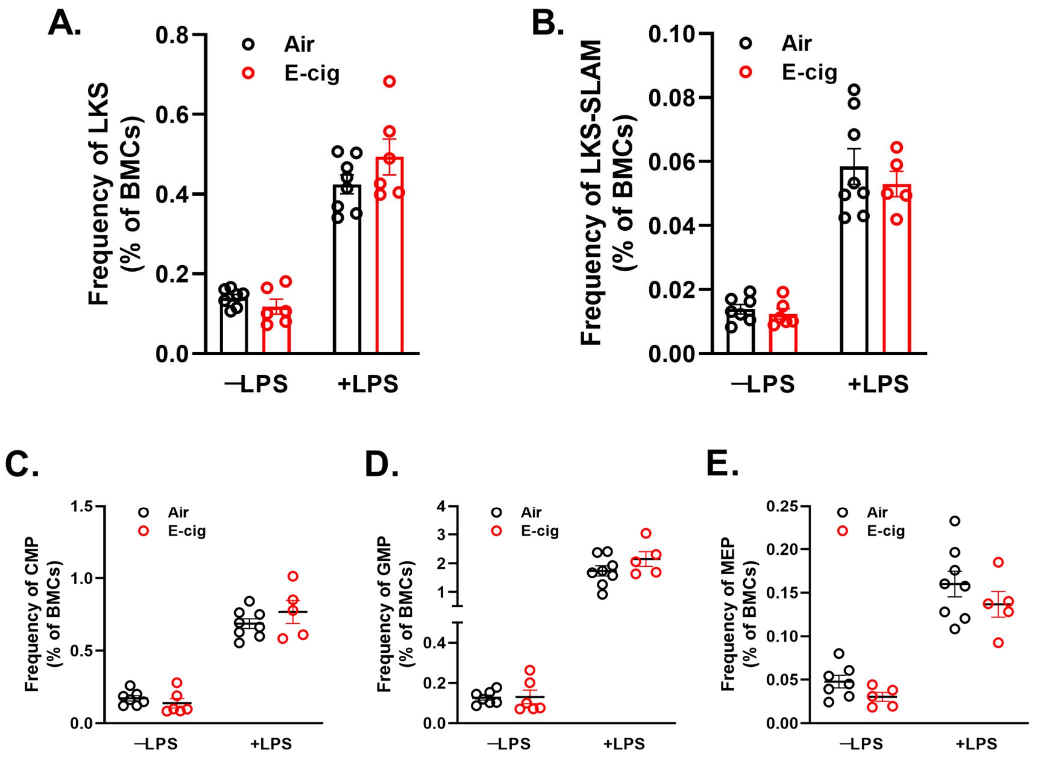

Figure S2. Systemic LPS increases HSPC populations in air and E-cigarette exposure groups. Bone marrow frequency of (**A**) LKS, (**B**) LKS-SLAM, (**C**) CMP, (**D**) GMP and (**E**) MEP populations are increased after LPS injection i.p. Data are shown as mean±SEM. *N* = 6–8 mice per group.

© 2020 by the authors. Licensee MDPI, Basel, Switzerland. This article is an open access article distributed under the terms and conditions of the Creative Commons Attribution (CC BY) license (http://creativecommons.org/licenses/by/4.0/).
